# Drug-induced hypersensitivity syndrome with high procalcitonin levels due to piperacillin/tazobactam and meropenem: A case report

**DOI:** 10.3389/fmed.2022.951714

**Published:** 2022-10-04

**Authors:** Gao Song, Meng-Qun Cheng, Rong Li, Cai-Qiong Zhang, Ping Sun

**Affiliations:** ^1^Department of Pharmacy, The Puer People's Hospital of Yunna City, Puer, China; ^2^Department of Reproductive Medicine, The Puer People's Hospital of Yunna City, Puer, China; ^3^Department of Science Education, The Puer People's Hospital of Yunna City, Puer, China

**Keywords:** β-lactam antibiotics, PCT, piperacillin/tazobactam, meropenem, DIHS, DRESS

## Abstract

Drug reaction with eosinophilia and systemic symptoms (DRESS) is a rare and life-threatening adverse drug reaction. It is characterized by a long latency period with rash, hematological abnormalities, and visceral damage. Clinical manifestations of DRESS vary. Thus, accurate clinical diagnosis and identification are essential to ensure timely treatment commencement for improving prognosis and speeding up recovery. We report the case of a 66-year-old male patient with a drug reaction induced by a beta-lactam antibiotic, piperacillin/tazobactam (Pip/Taz). This resulted in the manifestation of both eosinophilic and systemic symptoms. Ten days after the Pip/Taz treatment commencement, the patient developed hyperthermia and elevated serum procalcitonin (PCT), leading to a misdiagnosis of an exacerbated infection. Meropenem treatment was then started. However, after 72 h, the patient developed a generalized rash, eosinophilia, hematological abnormalities, and visceral damage. Moreover, PCT levels were significantly elevated. All these symptoms were associated with DRESS. The sensitizing drug was discontinued, and glucocorticoids were administered, resulting in gradual subsiding of symptoms and decreases in serum PCT levels. Clinicians should be aware that elevated PCT serum levels may be a diagnostic biomarker for DRESS, which requires specific treatment. Furthermore, studies are warranted to further evaluate and elucidate the role of PCT in response to DRESS.

## Introduction

Drug-induced hypersensitivity syndrome (DIHS), also known as drug reaction with eosinophilia and systemic symptoms (DRESS), is a rare and life-threatening adverse drug reaction. It is characterized by a long latency period with rash, hematological abnormalities, and visceral damage (1, 2). The mortality rate of DRESS is as high as 10% (3). Clinical symptoms of DRESS typically mimic those of sepsis. Moreover, it is often difficult to distinguish infectious diseases from DRESS due to their overlapping clinical manifestations. Such difficulties in differential diagnosis may raise concerns about misdiagnosis and unnecessary antibiotic therapy.

DRESS is caused by drug exposure, including anticonvulsants (namely, aromatase derivatives), antimicrobial agents (particularly, penicillin and sulfonamides), antipyretics, and analgesics (3). Up to 15–37% of DRESS syndrome cases may be caused by antibiotic administration (4). In a review of electronic health records in the United States between 1980 and 2016, antibiotics were attributed to DRESS syndrome in 74% of cases. These identified antibiotics included vancomycin (39%), beta-lactams (β-lactams) (23%), fluoroquinolones (4%), tetracyclines (4%), and sulfonamides (3%) (5).

Procalcitonin (PCT) is considered a specific biomarker for bacterial infection. However, previous studies have shown that PCT may be elevated in DRESS (6). The level of PCT elevation in patients with DRESS is inconclusive. However, it may have a prognostic value with PCT serum levels correlating with a higher incidence of DRESS severity (7). Antibiotic-associated DRESS with significantly elevated PCT was reported in a 6-year-old child due to cefotaxime and clindamycin use. However, that case improved with corticosteroid therapies (8).

This present case report describes an older man who developed DRESS, accompanied by high PCT levels following successive β-lactam piperacillin/tazobactam (Pip/Taz) and meropenem treatment, which was misdiagnosed as an exacerbated infection.

## Case description

**Figure 1:**
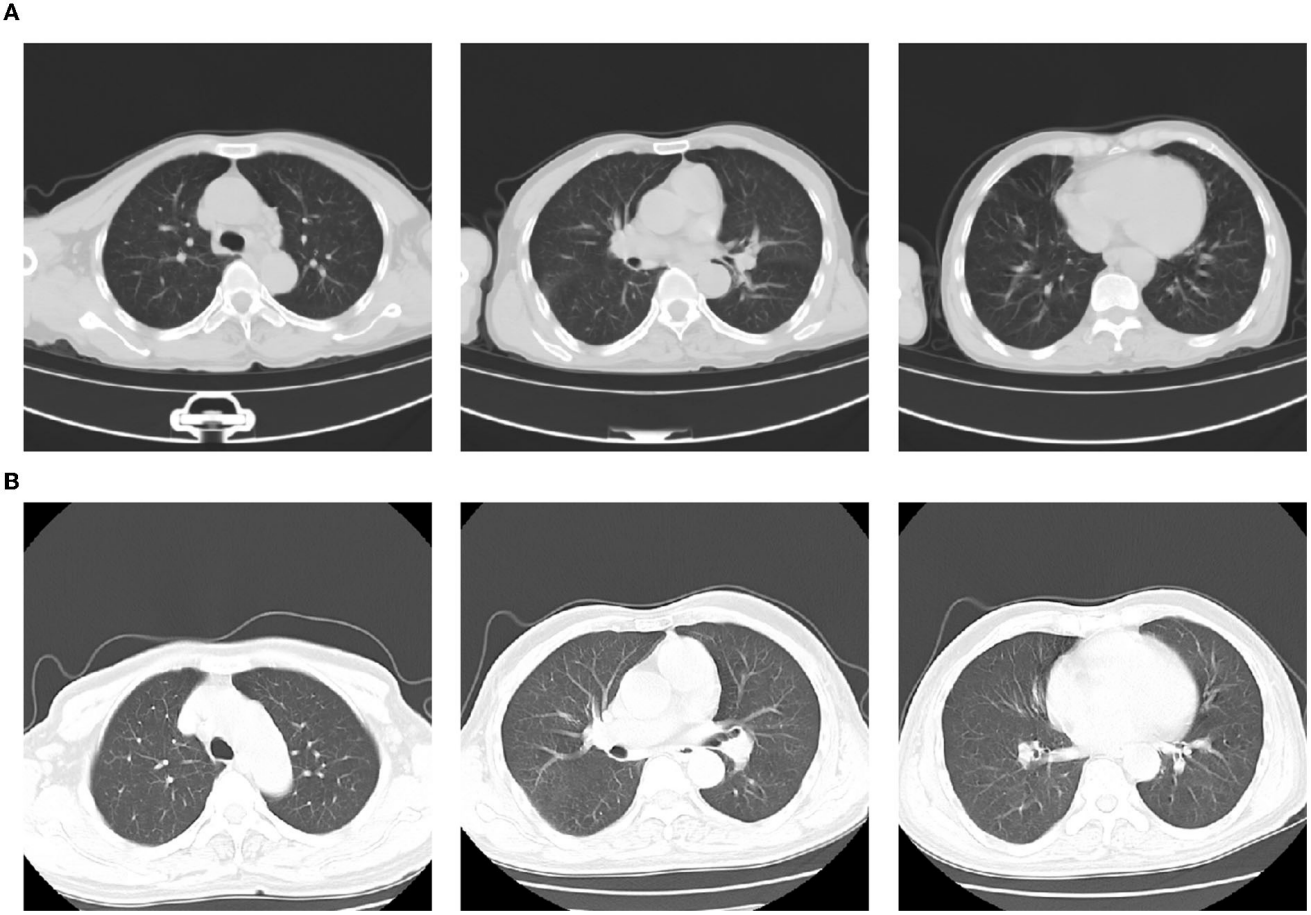
Computed tomography (CT) on admission showing chronic bronchitis with bilateral lung infection **(A)**; CT presentation on day 14 of hospitalization (day 3 of meropenem administration): No obvious lesions in both lungs. However, multiple enlarged lymph nodes are seen in the mediastinum and bilateral axillae **(B)**.

A 66-year-old male patient was admitted to the hospital with abnormal speech and behavior over 3 days. The patient had a history of hypertension and long-term heavy alcohol consumption (> 50 years). There was no history of liver disease. On admission, the patient had a low-grade fever (100.4°F). In addition, his other vital signs were stable. Physical examination revealed no generalized lymphadenopathy or hepatosplenomegaly. Neurological examination was unremarkable, and the patient was negative for meningism. Computed tomography (CT) of the chest revealed chronic bronchitis with bilateral lung infections (Figure 1). Abdominal CT showed no significant abnormalities. Screens for human immunodeficiency virus (HIV), syphilis, tuberculosis (TB) IgG antibodies, and hepatitis A, B, and C viruses were all negative. Cerebrospinal fluid (CSF) analysis showed no significant abnormality. Furthermore, culture results were negative. Laboratory test results showed an elevated white blood cell (WBC) count, 15.18 × 10^9^/L; high neutrophil percentage, 77%; and high urinary amylase, 1,118 U/L. His serum lactate dehydrogenase, liver transaminases, and bilirubin levels were normal.

**Figure 2:**
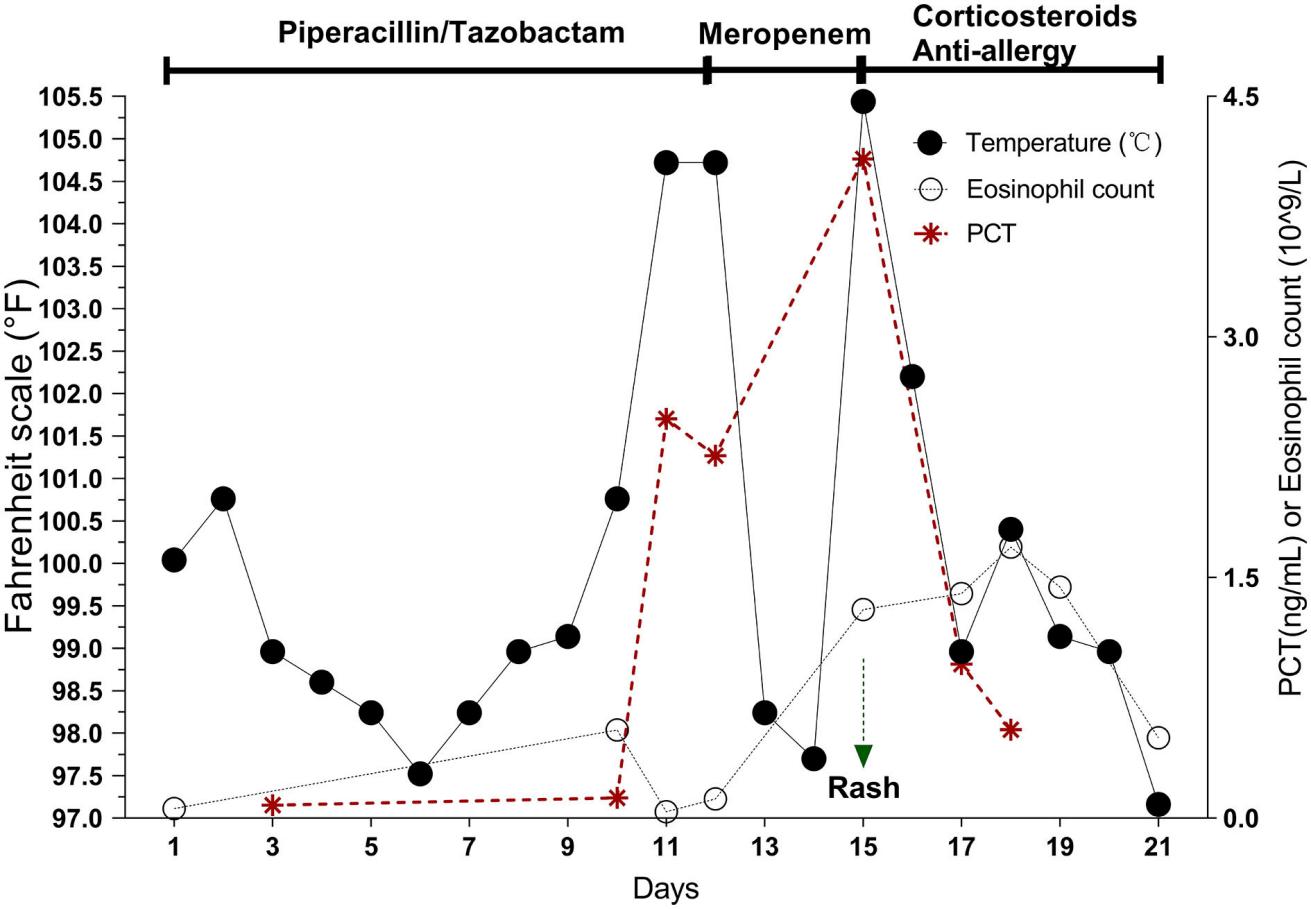
On days 10–12 of Pip/taz, the patient develops hyperthermia with persistently elevated PCT but normal eosinophils. After discontinuing Pip/taz and administering meropenem for 72 h (Day 15), the patient develops the characteristics of DRESS reaction (generalized rash, elevated eosinophils, hematologic abnormalities, and organ involvement), along with a markedly elevated PCT. After stopping the sensitizing drug and giving corticosteroids and anti-allergic drugs for about 1 week, the DRESS reaction subsides, and PCT decreases.

Pip/Taz 4.5 g intravenous (IV) q12h empirical anti-infective therapy was initiated on admission according to the Pip/Taz instructions of the “National Centralized Drug Procurement” policy (9). Treatment was effective. Seventy-two hours after treatment, the PCT level was at 0.08 ng/mL. The patient's respiratory symptoms improved, and his body temperature normalized. However, on day 10 after Pip/Taz treatment, the patient developed a fever of 104.7°F with chills and his PCT levels began to gradually increase. Therefore, we made dose adjustments based on the patient's response to treatment. Pip/Taz treatment was adjusted to 4.5g IV q8h. Additionally, blood, urine, and sputum samples were collected for analysis. Laboratory test results showed low WBC, 2.72 10^9^/L; normal neutrophil percentage, 48.8%; and high lymphocyte percentage, 43.8%, indicating relative lymphocytosis. Serum PCT was persistently elevated (Figure 2). D-dimer was 40.19 ug/mL. In addition, lactate dehydrogenase was high at 560 U/L. Serum levels of eosinophils, hepatic transaminase, bilirubin, and amylase, were all normal. The antibiotic treatment was switched to meropenem (1 g IV q8h), considering the possibility of exacerbated or drug-resistant bacterial infection.

Seventy-two hours after meropenem treatment, the patient again developed high a fever of 105.4°F. On examination, a generalized, pruritic non-blanching rash was observed. Although a chest CT scan showed no obvious lung lesions (Figure 1), it revealed multiple enlarged lymph nodes in the mediastinum and bilateral axillae. Blood culture results of blood, urine, and sputum specimens were all negative. A test for the protozoal parasite *Plasmodium vivax* was also negative. Laboratory test results showed high WBC, 15.9 × 10^9^/L; neutrophil percentage, 65.8%; and serum PCT, 4.11 ng/mL. These results also showed persistently elevated eosinophils,1.69 × 10^9^/L (Figure 2); B-type natriuretic peptide (BNP), 1,575 pg/mL; IL-6, 9.45 pg/ml; IL-10, 26.51 pg/ml; and interferon-gamma (IFN-γ), 116.17 pg/ml. Furthermore, cytomegalovirus IgG was elevated to 531 AU/mL. Levels of herpes simplex virus type I (HSV-I) IgG and herpes simplex virus type II (HSV-II) IgG were 339 AU/ml and 27 AU/ml, respectively. Serum creatinine, liver bilirubin, and transaminase levels were normal.

The patient experienced no significant discomfort during the fever. Several subspecialties assessed the patient for a non-infectious cause. Pip/Taz and meropenem-induced DRESS was considered based on elevated eosinophils, fever, almost universal non-blanching rash, lymphadenopathy, and organ involvement, all of which met DRESS diagnostic criteria (Table 1).

**Table 1:** Diagnostic criteria for potential drug reaction cases with eosinophilia and systemic symptoms (DRESS) published by the RegiSCAR (10).

Hospitalization
Reaction suspected to be drug-related
Acute rash
Fever > 100.4 °F*
Enlarged lymph nodes at a minimum of two sites*
Involvement of at least one internal organ*
Complete Blood count abnormalities*
Lymphocytes above or below normal limits
Eosinophils above the laboratory limits
Platelets below the laboratory limits

Meropenem treatment was discontinued, and methylprednisolone 500 mg IV daily was started for 3 days for shock therapy and tapered, along with concomitant anti-allergic treatment (specifically: intravenous calcium gluconate, oral ebastine, and topical calamine lotion). The patient's fever rapidly resolved, levels of eosinophils and PCT decreased and the generalized rash gradually subsided. However, BNP levels remained abnormal. Following discharge, the patient was prescribed prednisone (40 mg po daily for 2 weeks and then tapered over a 3-week period). Monthly telephone follow-ups were conducted. Five months following discharge, no further fever was reported, and the generalized rash was reported to have subsided.

## Discussion

In Asian countries, DRESS may account for 10% of adverse drug reactions (11). DRESS may show multiple clinical manifestations and systemic impairments, making differential diagnoses of infectious diseases, cancer, autoimmune diseases, and other disorders challenging, but essential. Clinically, diagnosis of DRESS is particularly challenging due to the absence of specific laboratory markers.

The pathogenesis of DRESS has not been fully elucidated. However, studies have shown that genetic polymorphisms and activation of human herpesviruses (HHV), specifically HHV 6/7, Epstein-Barr virus, and Cytomegalovirus (CMV), are associated with the pathogenesis of DRESS (1). Consequently, IgG activation of CMV, HSV-1, and HSV-II were monitored in this case study.

A long latency period is one of the characteristics of DRESS. Skin symptoms typically appear 2–6 weeks post inciting drug administration. Both fever and pruritus often occur several days before the patient's rash. Additionally, the body temperature may fluctuate between 100.4 and 104.0°F for several weeks. Early lesions are typically generalized measle-like macules or maculopapular rashes, which can be eczema-like or urticaria-like. In severe cases, lesions resemble exfoliative dermatitis and Stevens-Johnson syndrome (SJS) (12). Wang and Mei (13) showed that readministration of the same sensitizing drug could activate DRESS and exacerbate symptoms while shortening the latency period. The incubation period of carbapenem-inducing DRESS was almost the shortest (4 days) compared with other antimicrobial drugs (14). In this case, a similar phenomenon was observed. The patient developed prodromal fever symptoms on day 10 after treatment with Pip/Taz β-lactam antibiotic treatment. Meropenem is a β-lactam antibiotic treatment comprising a similar β-lactam ring structure to Pip/Taz. The patient's fever reappeared as intermittent hyperthermia (over 104°F) 72 h after switching Pip/Taz treatment to meropenem. This was accompanied by generalized rash and characteristic symptoms of DRESS.

DRESS can involve several different organ systems, most often the lymphatic, circulatory, and digestive systems, namely the liver. Lymph node involvement was seen in 75% of DRESS patients and manifests as either localized or generalized lymphadenopathy. Hematological involvement is mainly characterized by the increased number and proportion of leukocytes and eosinophils. Visceral damage is more likely to occur later than skin damage. However, in some patients, visceral damage can occur before skin lesions. The liver is the most commonly involved organ, and 75–94% of DRESS cases present hepatomegaly, elevated alanine aminotransferase, aspartate aminotransferase, and alkaline phosphatase. In some cases, this is accompanied by splenomegaly. Other involved organs include the kidney (12–40% of cases), lungs (1 in 3 cases), and heart (4–27% of cases).

In this case, DRESS involved the lymph nodes, hematological system, and heart, but not the liver. The results showed that at the first onset of hyperthermia on day 10, leukocytes were reduced. However, on day 15 following meropenem administration, the patient experienced fever again. Moreover, a persistent increase in eosinophils was observed. This is consistent with other studies in which patients may show decreases in leukocytes in the early stages with insignificant changes in eosinophils. However, eosinophil changes were significantly increased after 1–2 weeks following symptom onset (15).

There are no uniform diagnostic criteria for DRESS. The European and Japanese diagnostic criteria for DRESS are most widely used in clinical practice (10). The RegiSCAR European Registry of Serious Cutaneous Adverse Reactions (SCAR) evaluation system was used in this case. The SCAR system states that at least three of the four systemic features of DRESS (fever, lymphadenopathy, visceral organ involvement, and hematological abnormalities) must be present to meet SCAR DRESS diagnostic criteria (Table 1). In this case, the patient met these diagnostic criteria and could be diagnosed with DRESS.

**Figure 3:**
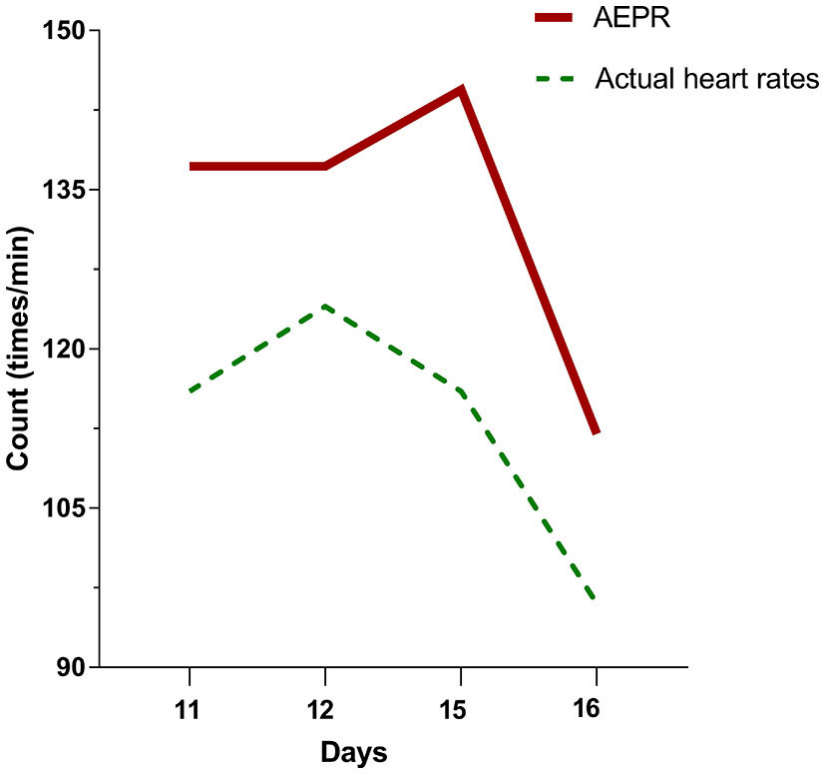
The patient's temperatures were above 102°F on days 11, 12, 15, and 16 of hospitalization. The actual heart rates of the patient at this time are all less than the approximate expected pulse response (AEPR). AEPR (times/minute) = [(temperature (°F) × 100) × 1] * 10 + 100.

In this case, we observed prodromal symptoms of hyperthermia with concomitant monitoring of drug fever accompanied by relative bradycardia (Figure 3). Allergic reactions are the most common cause of drug fever, especially in β-lactam antibiotics (16). The monitoring of relative bradycardia can discriminate the onset of drug fever in time to avoid further progression to DIHS.

PCT is increasingly used in clinical practice, primarily for diagnosing sepsis and bacterial infectious shock and guiding antibiotic therapy. PCT has a high specificity for detecting infection compared with other biomarkers, such as CRP or WBC. PCT concentrations > 0.5 ng/mL are considered consistent with bacterial infection. In addition, persistent PCT increases are recognized as a strong indicator of progressive systemic bacterial infection (17). In healthy participants, PCT concentration is < 0.1 ng/mL. However, in critically ill patients with non-infectious diseases, such as trauma, burns, myocardial infarction, cardiogenic shock, and malignancy, PCT is elevated, usually < 0.5 ng/mL as a result of major stressors causing systemic inflammation (18). PCT may also be elevated in anaphylaxis (19). However, induction of drug-specific T-cell activation in DRESS releases many cytokines and significantly increases the synthesis of various pro-inflammatory cytokines, namely, TNF-α, IL-5, IL-6, IL-2, and IFN-γ inducing PCT (20). A conclusive link to elevated serum PCT in patients with DRESS has not been elucidated due to a lack of data, with only a few studies reporting PCT values in DRESS cases (21). Said et al. (7) recorded PCT (ng/mL) in 91 patients diagnosed with DRESS without bacterial infection. Results showed that 31.9% of patients had elevated serum PCT >0.5 ng/mL. In this case, infectious diseases and other false positive PCT elevation factors were ruled out. On days 10–12, with initial hyperthermia onset, PCT began to rise persistently. On day 15, the second onset of hyperthermia occurred with DRESS reaction. Herein, PCT increased to 4.11 ng/mL, exceeding the threshold value of infection (0.5 ng/mL) by more than 8-fold. When we discontinued the sensitizing drug, PCT decreased significantly, which further ruled out evidence of bacterial infection. Due to the high negative predictive value of PCT, its role in ruling out bacterial infections may be even greater (22).

Treatment of DRESS should be individualized (12, 15). Moreover, a treatment protocol should be chosen according to the severity of visceral organ involvement. First, sensitizing drugs should be discontinued. In addition, topical glucocorticoids and supportive therapy should be administered to patients with mild-to-moderate disease. In severe cases of multiple organ involvement, such as the liver, kidney, heart, and lungs, systemic glucocorticoids should be administered. When reactivation of the associated virus is confirmed, antiviral drugs such as ganciclovir can be added. Said et al. (7) reported that patients with elevated PCT levels had more severe clinical DRESS manifestations with poor prognoses. In this study, the patient had organ involvement and a significantly elevated PCT level. Therefore, anti-allergic and systemic corticosteroid therapies were administered along with prompt discontinuation of the sensitizing β-lactam drugs. After treatment, follow-up and laboratory measures confirmed the patient's DRESS-related symptoms were effectively controlled. The patient's prognosis was good, with no recurrence of symptoms at either 1- or 5-month follow-up after discharge.

## Conclusion

In conclusion, clinicians should be aware that elevated PCT serum levels may be a diagnostic biomarker for DRESS requiring specific treatment. Furthermore, additional studies should be conducted to further evaluate and elucidate the role of PCT in response to DRESS. To the best of our knowledge, this is the first case report of β-lactam antibiotic, Pip/Taz, and meropenem-induced DRESS associated with significant PCT elevation.

## Data availability statement

The original contributions presented in the study are included in the article/supplementary material, further inquiries can be directed to the corresponding authors.

## Ethics statement

Ethical review and approval was not required for the study on human participants in accordance with the local legislation and institutional requirements. The patients/participants provided their written informed consent to participate in this study. Written informed consent was obtained from the individual(s) for the publication of any potentially identifiable images or data included in this article.

## Author contributions

GS, RL, and C-QZ were involved in the clinical management of the patient. GS and RL contributed to the diagnosis in this study. GS integrated all information and wrote the manuscript. GS, PS, and M-QC participated in the follow-up process. M-QC provided significant guidance and revisions to GS throughout the writing process. All authors contributed to the article and approved the submitted version.

## Funding

This work was supported by the Yunnan Province, Kunming Medical University Joint Special (202201AY070001-294).

## Conflict of interest

The authors declare that the research was conducted in the absence of any commercial or financial relationships that could be construed as a potential conflict of interest.

## Publisher's note

All claims expressed in this article are solely those of the authors and do not necessarily represent those of their affiliated organizations, or those of the publisher, the editors and the reviewers. Any product that may be evaluated in this article, or claim that may be made by its manufacturer, is not guaranteed or endorsed by the publisher.
